# The relative toxicity of medicines detected after poisoning suicide deaths in Australia, 2013–19: a data linkage case series study

**DOI:** 10.5694/mja2.52638

**Published:** 2025-03-24

**Authors:** Jessy Lim, Nicholas A Buckley, Kate Chitty, Andrea L Schaffer, Jennifer Schumann, Zein Ali, Rose Cairns

**Affiliations:** ^1^ The University of Sydney Sydney NSW; ^2^ New South Wales Poisons Information Centre the Children's Hospital at Westmead Sydney NSW; ^3^ The University of Western Australia Perth WA; ^4^ The Bennett Institute of Applied Data Science University of Oxford Oxford United Kingdom; ^5^ The Victorian Institute of Forensic Medicine Monash University Melbourne VIC; ^6^ Monash Addiction Research Centre Monash University Melbourne VIC

**Keywords:** Suicide, Overdose, Forensic medicine, Mental disorders, Prescription drugs, Toxicology

## Abstract

**Objective:**

To compare the toxicity (relative to population use) and lethality (relative to poisoning events) of medicines involved in poisoning suicides in Australia; to determine the proportions of cases in which the medicines had recently been dispensed to the deceased person.

**Study design:**

Case series study; analysis of linked National Coronial Information System (NCIS) and Pharmaceutical Benefits Scheme (PBS) data.

**Setting, participants:**

Closed coronial cases for deaths of people aged ten years or older deemed to have been medicine poisoning suicides (including multiple cause deaths), Australia, 1 July 2013 – 10 October 2019, with recorded post mortem toxicology findings.

**Main outcome measures:**

Fatal toxicity index (FTI): deaths per million years of use at the defined daily dose in Australia (2013–2015); proportion of FTI attributable to medicines dispensed to the deceased person during the twelve months preceding their death; estimated case fatality: deaths per number of calls to poisons information centres regarding the medicine (based on the number of calls to the NSW Poisons Information Centre, 2013–2017).

**Results:**

During 2013–19, 2132 deaths were classified as medicine poisoning suicide deaths (median age, 51 years [interquartile range, 39–64 years]; 1036 girls or women [49%]). The 5703 detected substances deemed to have contributed to death included 140 medicines. The overall FTI was 32.0 (95% confidence interval [CI], 30.6–33.3) deaths per million years of use; overall estimated case fatality was 1.28% (95% CI, 1.23–1.34%) of poisoning events. FTI and estimated case fatality (each log_10_ transformed) were moderately correlated (*R* ^2^ = 0.66). Both values were relatively high for most opioids, sedative psychotropics, and tricyclic antidepressants. Specific medicines with high values were phenobarbitone, oxycodone, morphine, clonazepam, nortriptyline, and propranolol; they were relatively low for risperidone and lithium. The proportions of opioids and hypnosedatives that had been recently dispensed to the deceased persons were smaller than for antidepressant, antipsychotic, and antiepileptic medicines.

**Conclusions:**

To reduce the risk of suicide, access to medicines of greater toxicity and lethality should be restricted, including by staged supply (regular supply of medicines in limited quantities), and limiting pack sizes; real‐time prescription monitoring could detect and minimise stockpiling.

**The known**: Poisoning with medicines is a frequent suicide method in Australia. Means restriction is an evidence‐based suicide prevention strategy, and should be guided by the relative toxicity of medicines.

**The new**: Opioids, hypnosedatives (phenobarbitone and benzodiazepines), tricyclic antidepressants, and propranolol were more frequently involved in suicide deaths than expected from their overall population use; their lethality in cases of poisoning was also relatively high. Risperidone and lithium were the least toxic and lethal psychotropic medicines.

**The implications**: The least toxic options in a drug class should be considered first when prescribing medicines. More toxic medicines should be supplied in limited quantities and their dispensing to individuals monitored.

Means restriction as a suicide prevention strategy is supported by strong evidence.^1^, ^2^ Restricting public access to hazardous poisons reduces the frequency of suicides by poisoning without a compensatory increase in other suicide methods.^2^, ^3^ Medicines are accessible poisons often used in suicide attempts, particularly impulsive attempts.^4^ Characterising the role of medicines in poisoning suicides is complicated, as they are also detected in people who have died by other means. Sedatives, opioids, and drug combinations are more likely to be detected after poisoning suicides in Australia than after other types of suicide.^5^

Medicines implicated in poisoning suicides are often widely available in the community.^6^ The fatal toxicity index (FTI) and case fatality are measures of the toxicity of a medicine relative to its population‐level use. FTI is the number of poisoning deaths divided by a measure of population use.^7^, ^8^, ^9^ Case fatality, a measure of the lethality of an overdose, is the number of poisoning deaths divided by the number of poisoning events.^6^, ^10^ FTI and case fatality studies in North America, Europe, and Australia and New Zealand have found that psychotropic agents, particularly opioids, hypnosedatives, and antidepressants, are the medications most frequently involved in poisoning deaths.^6^, ^7^, ^8^, ^9^, ^10^ Case fatality is also high for some medicines used for treating diabetes or cardiovascular conditions.^10^ FTI and case fatality are generally correlated.^9^

FTI studies have not reported whether medicines that contributed to suicide deaths had been dispensed to the deceased persons. Linking the medicine dispensing histories of individuals with poisoning outcomes could provide insights into medicines obtained and subsequently used for intentional self‐poisoning.^11^ Closer control of more frequently dispensed medicines could include suicide risk assessment by clinicians, supplying smaller quantities, and monitoring dispensing habits.^12^ The implication of medicines that are dispensed less frequently could indicate old prescriptions, use of another person's medicine,^11^ or diversion.^13^

In this study, we investigated the medicines implicated in medicine poisoning suicides in Australia during 2013–19. We assessed their relative toxicity and lethality as FTI and estimated case fatality, and used data linkage to determine the proportions of cases in which medicines had been dispensed to each deceased person during the twelve months preceding their death.

## Methods

Our study was part of the larger Australian Suicide Prevention using Health Linked data (ASHLi) study, a population‐based case series study of all people who died by suicide in Australia during 1 July 2013 – 10 October 2019. The ASHLi study examined coronial cases for deaths of people in Australia aged ten years or older that had been closed by the coroner and deemed to be the result of intentional self‐harm.^11^, ^14^

We extracted post mortem toxicology data for people who died by suicide in Australia from the National Coronial Information System (NCIS) database. Sex is classified by the NCIS according to biological characteristics at the time of death. If the cause of death involved a combination of methods, such as poisoning and hanging, we deemed it a poisoning‐related suicide. Suicides were classified as medicine or drug poisoning suicides, non‐therapeutic gas or chemical poisoning suicides, or non‐poisoning suicides; this article is a case series study of medicine or drug poisoning suicides.

Coroners’ findings were reviewed to identify which detected substances were deemed to have contributed to the death. A substance was considered contributory if it was specifically mentioned by the coroner in the cause of death free‐text fields, or it matched an International Statistical Classification of Diseases, tenth revision (ICD‐10) code for poisoning exposure in the coded ICD‐10 fields (Supporting Information, table 1). Metabolites were not considered separately if the parent drug was also detected (Supporting Information, table 2).

### Population‐level medicines use

The Pharmaceutical Benefits Scheme (PBS) subsidises medicines for Australian citizens and other eligible recipients.^15^ The PBS database captures about 75% of prescription medicine use in Australia; It does not capture inpatient public hospital medicine use, over‐the‐counter medicines, or medicines dispensed on private prescriptions.^16^ Medicines dispensed on private prescriptions do not meet PBS subsidy criteria (eg, off‐label use), or are priced outside the PBS‐recommended price range.^16^

The PBS Statistics on Medicines reported medicine use as defined daily dose (DDD) per 1000 population per day, based on the mean standard daily dose of each drug for its main indication.^15^ The DDD/1000 population/day is consequently a proxy measure of the total number of people taking the DDD each day.^15^ We calculated the mean DDD/1000 population/day for each medicine in Australia for the calendar years 2013, 2014, and 2015 (the final three years of the Statistics on Medicines dataset).

### Individual linked medicine dispensing history

Post mortem toxicology findings for each individual were merged with their PBS medicine dispensing history by data linkage. We deemed a medicine recently dispensed if it had been dispensed within twelve months of the date of death.

### Poisons information centre calls

The New South Wales Poisons Information Centre (NSWPIC) receives about 50% of poisoning‐related calls in Australia, including calls from other states.^17^ We extracted the number of calls regarding intentional self‐poisoning exposures for each medicine in the NSWPIC database during 1 January 2013 – 31 December 2017; we excluded second or further calls regarding an exposure. This call period was selected because reporting lag means that complete suicide data for 2018 and 2019 were not available (1830 of the 2132 included suicide deaths [86%] were during 2013–2017).^5^, ^14^ We multiplied call numbers by two to estimate the national figure (adjusted number of calls).

### Outcomes

The specific outcomes of our study were:

- FTI values for medicines that contributed to death in medicine poisoning suicides, relative to population use;
- the FTI proportions for medicines that had or had not recently been dispensed to the deceased person;
- the estimated case fatality for medicines that contributed to death in medicine poisoning suicides, relative to the number of poisoning events (poisons information centre calls); and
- graphic classification of medicines that contributed to death in medicine poisoning suicides according to their toxicity and lethality.

### Data analysis

As more than one substance can contribute to a death, we calculated weighted frequencies for each contributory substance; for example, if five substances contributed to a death, each was assigned a weighted value of 0.2. The weighted values were summed to calculate the total weighted frequency for a medicine; 95% confidence intervals (CIs) were estimated using the Poisson distribution.^18^

We included substances in our analysis if they appeared in the PBS list of medicines with DDDs, or NSWPIC calls about intentional self‐poisoning involving the substances were recorded. Illicit drugs and non‐medicines were assigned weighted frequencies but were not included in our analysis.

We calculated the FTI value for each medicine. The numerator was the weighted frequency for the medicine; the denominator was the DDD/1000 population/day × the 2015 Australian population size (23.8 million)^19^ × five (to estimate the number of people using the medicine over five years). We report the FTI as numbers of deaths per million years of use, with 95% CIs estimated by dividing the upper and lower CI values of the weighted frequency by the same denominator. We excluded medicines for which accurate DDD values were unlikely, including those typically used for resuscitation in hospitals or by paramedics, and over‐the‐counter medicines. We also excluded medicines with very low DDD values (lowest fifth percentile), including medicines which are subsidised only by the Repatriation Pharmaceutical Benefits Scheme or require an authority prescription with specific criteria (Supporting Information, box 1).

Box 1 **Selection of medicines implicated in suicide deaths in Australia, 1 July 2013 – 10 October 2019, for the calculation of fatal toxicity index (FTI) values and the estimation of case fatality**

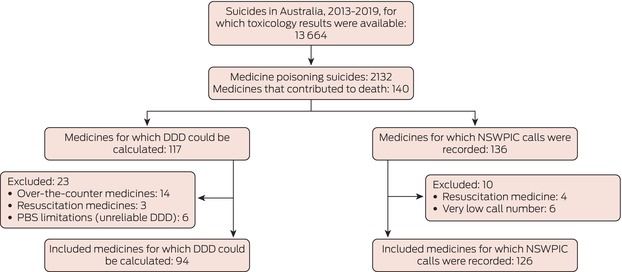

DDD = defined daily dose; NSWPIC = New South Wales Poisons Information Centre; PBS = Pharmaceutical Benefits Scheme.

We also recalculated the FTI for each medicine, stratified by whether PBS‐subsidised dispensing of the medicine to the deceased person during the twelve months preceding their death was recorded (recently dispensed medicines) or it was not (non‐dispensed medicines).

Given the frequency of their use in the community, we also calculated FTI values for nervous system medicines (Anatomical Therapeutic Classification^20^) not detected in suicide deaths, but for which published DDD values were available.

We also estimated case fatality for each medicine, by dividing its weighted frequency by the adjusted number of intentional self‐poisoning calls to the NSWPIC; 95% CIs were estimated by dividing the upper and lower CI values of its weighted frequency by the same denominator. Not all poisoning events reported to poisons information centres lead to hospital admissions; NSWPIC, for example, receives calls from the public and emergency departments. Conversely, not all poisonings result in calls to poisons information centres. The NSWPIC database captures a larger number of intentional self‐poisonings than alternative data sources, such as paramedic records and hospital admissions; 28% of calls to Australian poisons information centres are from health care professionals.^21^ We estimated case fatality for over‐the‐counter medicines, but not for resuscitation medicines and medicines with very low NSWPIC call numbers (lowest fifth percentile). We estimated case fatality from poisons information centre cells data, but most case fatality studies are based on hospital admission records.^10^, ^22^

Finally, we graphed estimated case fatality against FTI for medicines for which both values were available.

Analyses were conducted in SAS 9.3 and Microsoft Excel.

### Ethics approval

The ASHLi study was approved by the Victorian Department of Justice and Community Safety human research ethics committee (CF/17/23250), the Western Australian coronial ethics committee (EC14/2018), the Australian Institute of Health and Welfare ethics committee (EO2017/4/366), and the NSW Population and Health Services research ethics committee (2017/HRE1204).

## Results

Toxicology results were available in the NCIS for 13 664 suicide deaths during 2013–19 (median age, 44 years; interquartile range [IQR], 31–57 years; 3314 girls or women [24%]), of which 2132 were classified as medicine poisoning suicides (median age, 51 years [IQR, 39–64 years]; 1036 girls or women [49%]) (Box 1). Of 10 530 substances identified in the toxicology reports, 5703 substances were deemed to have contributed to death (mean number per person, 2.65 [standard deviation, 1.96]; median, 2 [IQR, 1–4]), including 140 medicines (Box 2). The total DDD for all medicines included in FTI calculations was 560 DDDs/1000 population/day (66.7 million years of use over five years); the adjusted number of 166 554 poisoning calls was used to estimate case fatality. The overall FTI was 32.0 (95% CI, 30.6–33.3) deaths per million years of use; overall estimated case fatality was 1.28% (95% CI, 1.23–1.34%) of poisoning events.

Box 2 **Numbers of contributory substances identified by toxicology analysis in 2132 medicine poisoning suicide deaths, Australia, 1 July 2013 – 10 October 2019**

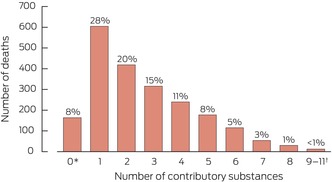

* Substance could not be assayed or detected, but determined cause of death was based on circumstantial evidence; double occurrence of insufficient or missed data (vague statements by coroner and International Statistical Classification of Diseases, tenth revision code not provided); decomposition or other factors affected feasibility of forensic testing.

† Combined to avoid depicting individual values below six.

### Fatal toxicity index

After excluding resuscitation and over‐the‐counter medicines and those with very low DDD values (Supporting Information, table 3), we calculated the FTI for 94 medicines. The medicines with the highest FTI values (deaths per million years of use) were clonazepam (1592; 95% CI, 1020–2368), phenobarbitone (677; 95% CI, 279–1429), oxycodone (365; 95% CI, 309–431), quetiapine (268; 95% CI, 215–332), morphine (241; 95% CI, 163–344), and chlorpromazine (225; 95% CI, 69.6–654). FTI values were higher for tricyclic antidepressants, particularly nortriptyline (214; 95% CI, 101–421) and clomipramine (211; 95% CI, 77.3–459), than for other antidepressants; the highest value for a non‐psychotropic medicine was for propranolol (204; 95% CI, 139–293) (Box 3; Supporting Information, table 4).

Box 3 Fatal toxicity index (FTI) values for the fifty prescription medicines implicated in medicine poisoning suicide deaths with highest FTI values*

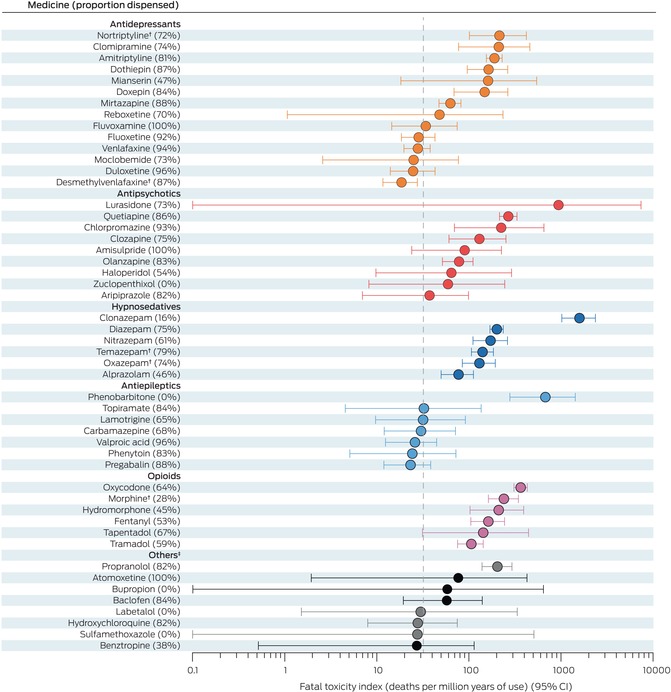

CI = confidence interval.

* The FTI values for all 94 medicines for which FTI values were calculated are provided in the Supporting Information, table 4. The weighted value of each medicine was adjusted according to the number of medicines deemed to contribute to each death. “Proportion dispensed” refers to medicines dispensing to the deceased person during the twelve months preceding their death, as recorded in the Pharmaceutical Benefits Scheme database. The vertical dotted line is overall FTI for all 2132 medicine poisoning suicide deaths (32.0 deaths per million years of use; 95% CI, 30.6–33.3 deaths per million years of use).

† Included only if parent compound was not also detected (Supporting Information, table 2).

‡ Dark circles: psychotropic medicines; grey: non‐psychotropic medicines.

We successfully linked NCIS data for 2040 of 2132 people (96%) with their PBS records. The dispensed medicine FTI proportions were larger for antidepressants (mean recently dispensed rate, 86%), antipsychotics (77%), and antiepileptics (61%) than for opioids (53%) and hypnosedatives (59%); the dispensed medicine FTI proportion was 64% for oxycodone, zero for phenobarbitone, and 16% for clonazepam (Box 3; Supporting Information, table 4). The FTI values for nervous system medicines that were not detected and had not contributed to deaths had published FTI values of zero (Supporting Information, table 5).

### Estimated case fatality

After excluding resuscitation medicines and medicines with very few poisons information centre calls (Supporting Information, table 6), we estimated case fatality for 126 medicines. The medicines with the largest values were opioids (dextropropoxyphene: 23.3% [95% CI, 7.4–53.0%]; fentanyl: 12.3% [95% CI, 7.9–18.4%]; hydromorphone: 12.3% [95% CI, 6.0–23.0%]; morphine: 10.3% [95% CI, 6.9–14.7%]) and phenobarbitone (12.2% [95% CI, 5.0–25.8%]). Among tricyclic antidepressants, estimated case fatality was highest for doxepin (7.3%; 95% CI, 3.4–13.0%), dothiepin (7.1%; 95% CI, 4.2–11.4%), and nortriptyline (5.6%; 95% CI, 2.7–11.1%); among antipsychotics, it was highest for zuclopenthixol (8.0%; 95% CI, 1.1–32.8%) and clozapine (2.9%; 95% CI, 1.4–5.6%); among cardiovascular drugs, it was highest for diltiazem (6.2%; 95% CI, 2.6–13.4%), flecainide (4.3%; 95% CI, 0.1–23.2%), and verapamil (3.8%; 95% CI, 1.3–9.6%). The over‐the‐counter medicines with highest estimated case fatality were codeine (4.7%; 95% CI, 3.8–5.8%), salicylic acid (3.5%; 95% CI, 0.6–8.8%), pholcodine (3.1%; 95% CI, 0.4–10.3%), and diphenhydramine (1.3%; 95% CI, 0.2–4.8%) (Box 4; Supporting Information, table 7).

Box 4 Estimated case fatality for the fifty medicines implicated in medicine poisoning suicide deaths with highest case fatality values*

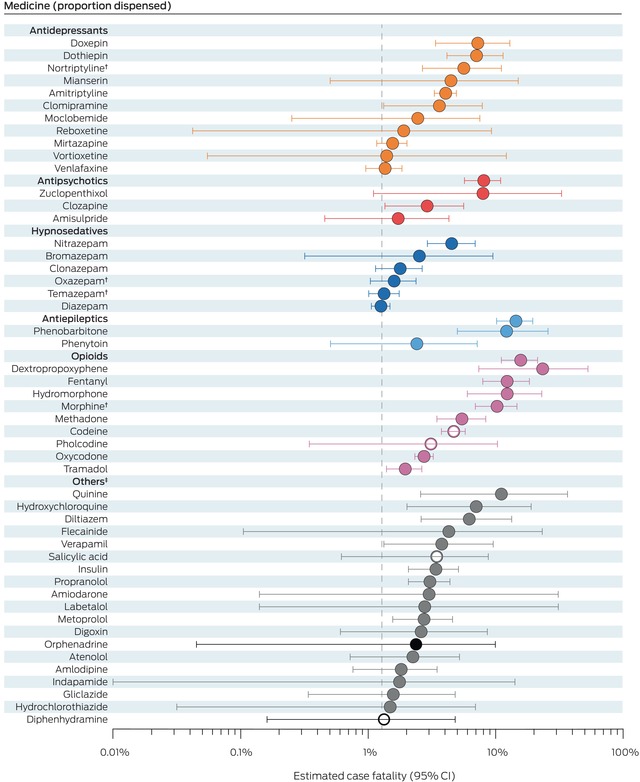

CI = confidence interval.

* The estimated case fatality for all 126 medicines for which FTI values were calculated are provided in the Supporting Information, table 7. The weighted value of each medicine was adjusted according to the number of medicines deemed to contribute to each death. Open circles indicate four medicines that are available over the counter or without prescription; fatal toxicity index values could not be estimated for these medicines. The vertical dotted line is overall estimated case fatality for all 2132 medicine poisoning suicide deaths (1.28%; 95% CI, 1.23–1.34%).

† Included only if parent compound was not also detected (Supporting Information, table 2).

‡ Dark circles: psychotropic medicines; grey: non‐psychotropic medicines.

### Correlation of fatal toxicity index and estimated case fatality

For all medicines for which we report both values, FTI and estimated case fatality (each log_10_ converted) were moderately correlated (*R* ^2^ = 0.66) (Supporting Information, figure 3). FTI values and estimated case fatality were both relatively high for tricyclic antidepressants, mianserin, clonazepam, nitrazepam, phenobarbitone, and propranolol, particularly in comparison with other medicines from their respective drug classes; both values were high for most opioids (Box 5).

Box 5 Fatal toxicity index (FTI) and estimated case fatality for the 97 medicines implicated in medicine poisoning suicide deaths for which both values were available, by drug class*

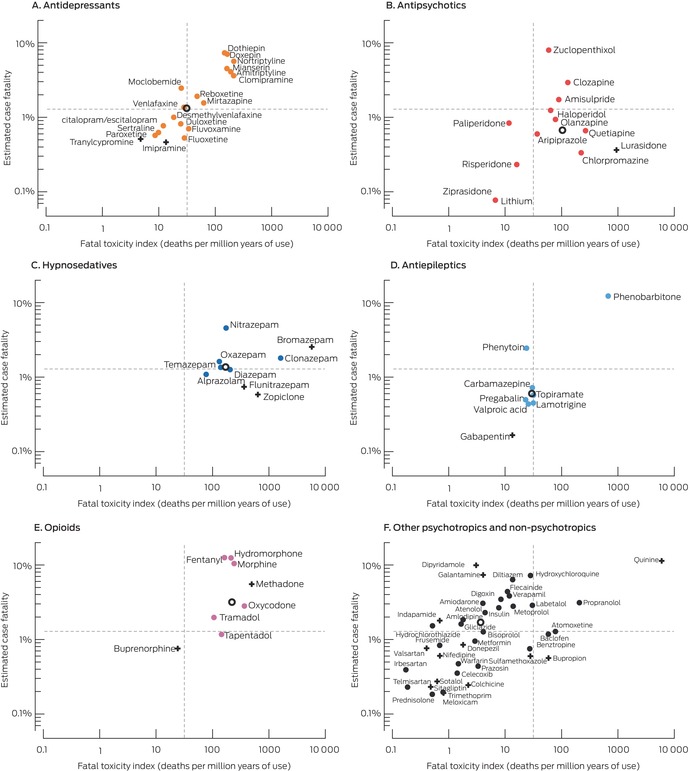

* The dotted lines indicate the overall FTI and estimated case fatality for all 2132 medicine suicide deaths. Resuscitation and over‐the‐counter medicines are not depicted; results for medicines with unusual dispensing patterns (Pharmaceutical Benefits Scheme limitations, very small numbers) or statistically non‐significant 95% confidence intervals for FTI or estimated case fatality should be interpreted with caution (indicated by +). The overall FTI and estimated case fatality for a drug group (for medicines for which both values were available) are indicated by an open circle.

## Discussion

Of the medicines frequently implicated in poisoning suicides in Australia, we found that the risks of toxicity relative to population use and lethality in cases of poisoning are particularly high for opioids, hypnosedatives, tricyclic antidepressants, and propranolol. Other studies of suicide have similarly reported that the frequency of involvement, toxicity, and lethality are highest for opioids, hypnosedatives, and sedative psychotropic medicines.^5^, ^7^, ^9^, ^10^, ^23^ FTI values were relatively high for tricyclic antidepressants (other than imipramine) and mianserin. The authors of other FTI studies have recommended that tricyclic antidepressants, venlafaxine, and citalopram be prescribed as antidepressants with caution;^7^, ^8^, ^24^ the FTI values for mirtazapine and reboxetine in our study were higher than that for venlafaxine. Among selective serotonin reuptake inhibitors (SSRIs), the highest FTI values were for fluvoxamine and fluoxetine; the estimated case fatality was highest for citalopram and escitalopram. Among antipsychotics, the highest FTI values were for quetiapine and chlorpromazine; the estimated case fatality was highest for zuclopenthixol and clozapine. FTI values for risperidone and lithium were relatively low. Other studies have identified clozapine as the antipsychotic with the highest FTI; both high and low FTI values for quetiapine and lower FTI values for lithium, risperidone, and antiepileptics (as mood stabilisers) have been reported.^7^, ^8^, ^9^, ^25^

The frequent involvement of psychotropic medicines in poisoning suicides is unsurprising, as they are accessible to people at risk of suicide.^11^, ^12^ The risk of toxicity associated with specific psychotropic agents is considered by prescribing guidelines. SSRIs are recommended as first‐line antidepressants because the risk of toxicity, especially in overdose, is lower than for tricyclic antidepressants.^24^, ^26^ Second generation antipsychotics are generally less toxic in overdose than other antipsychotics, particularly risperidone, paliperidone, and aripiprazole,^27^ which are available in long‐acting depot injection formulations that minimise the risk of intentional overdose.^28^ Lithium should also be considered for its efficacy in reducing suicidal behaviour and its low toxicity.^25^

When psychotropic agents associated with greater risk of toxicity are prescribed for complex conditions (eg, treatment‐resistant depression or schizophrenia), the potential for stockpiling should be reduced by mechanisms such as real‐time prescription monitoring or staged supply.^5^, ^29^ Prescribing limited quantities (ie, the smallest practical pack size, including partial or broken packs) could protect both the intended user and any household members with access to their medicines.^30^ Risk assessment is also warranted for non‐psychotropic use of these agents, particularly tricyclic antidepressants, which are used for treating neuropathic pain and migraine at higher rates of dispensing that could be monitored.^31^

Phenobarbitone, with a relatively high FTI in our analysis, and barbiturates are still often implicated in poisoning suicides.^5^, ^10^, ^32^ However, none of the fourteen deaths in which phenobarbitone was implicated in our study were linked with its PBS‐subsidised dispensing, which implies that it was obtained without prescription or used for indications other than PBS‐approved epilepsy. This finding could be related to the promotion and online sale of barbiturates for use in suicide.^32^ The proportions of recently dispensed medicines frequently implicated in suicide were also lower for hypnosedatives and opioids than overall, which may reflect diversion, stockpiling, private prescriptions, or using medicines prescribed for other household members.

FTI values were relatively high for opioids, including oxycodone, fentanyl, morphine, and hydromorphone. As methadone and buprenorphine supplied in hospitals or clinics for opioid substitution therapy were not recorded in the PBS database during the study period,^15^ we may have underestimated their prescribed use. Buprenorphine was the least toxic opioid in our study, possibly because it is a partial opioid agonist, and because transdermal preparations are frequently used.^33^ In contrast, the FTI for methadone was relatively high, as also reported by other studies.^6^, ^7^, ^8^, ^9^

The frequent involvement and relatively high lethality of calcium channel blockers (especially diltiazem and verapamil) and propranolol in poisoning suicides have also been reported in other studies.^5^, ^10^, ^23^ When prescribing non‐psychotropic medicines such as propranolol, physicians should consider assessing the suicide risk of the patient.

Among the over‐the‐counter medicines we assessed, estimated case fatality was relatively high for antitussives and sedating antihistamines, medicines that are misused for recreational purposes or sedation.^34^, ^35^ Diphenhydramine was the over‐the‐counter medicine most frequently implicated in overdose suicides in Toronto, Canada.^23^ Up‐scheduling or real‐time recording of supply could reduce harm from these medicines. The estimated case fatality of codeine was relatively high; it was re‐scheduled during the study period (in 2018) from over‐the‐counter to prescription only, and the number of self‐poisonings has subsequently declined.^36^ Sales of dextropropoxyphene‐containing products were discontinued entirely in 2018.^37^

### Limitations

Our FTI calculations were based on PBS medicines dispensing data. We may have overestimated the FTI for items with restricted subsidisation and those that are often dispensed on private prescriptions, including quinine, zopiclone, bromazepam, and flunitrazepam. FTI values for bromazepam and flunitrazepam were high, but no suicide deaths involved these agents as medicines dispensed to the deceased person; they may have been taken as illicit or diverted benzodiazepines, or prescribed on private prescriptions.

Australian DDD data have not been published after 2015. This may affect the FTI for medicines such as pregabalin, for which the prescribing criteria changed several times during the study period.^38^ Further, the DDD refers only to the main indication for a medicine. Frequent off‐label low dose (25 mg) use of quetiapine as a sleep aid, for example, could consequently lead to underestimating the number of users.^39^

Estimated case fatality was based on calls to NSWPIC about intentional self‐poisonings. However, not all poisonings lead to calls, and people may be more likely to call about substances they know to be toxic, such as tricyclic antidepressants. We extrapolated the number of NSWPIC calls to Australia as we did not have calls data for other poisons information centres. The ranking of our case fatality estimates was similar to those of case fatality studies based on hospital admissions data.^16^ Data for some medicines with very high FTI values or estimated case fatality were limited, and should be interpreted with caution.

Toxicology analysis detected alcohol in 4318 of 13 664 suicide cases (32%).^5^ Risky alcohol use is associated with suicide and can increase the toxicity of poisoning combinations, especially sedative medicines.^5^, ^10^ We included mixed methods of suicide, such as hanging with poisoning (259 of 2132 included cases, 12%). While all medicines in our study were coded in the NCIS database as contributing to death, they may have been less important than other non‐medicine substances or suicide methods.

### Conclusions

Of the medicines detected in poisoning suicides in Australia during 2013–19, measures of toxicity and lethality were highest for opioids, sedative psychotropics, and tricyclic antidepressants; phenobarbitone, oxycodone, morphine, clonazepam, and nortriptyline were ranked particularly high on these measures, as was propranolol. In contrast, the toxicity of risperidone and lithium were relatively low. To reduce the risk of suicide, access to medicines of greater toxicity and lethality should be restricted, including by staged supply (regular supply of medicines in limited quantities) and limiting pack sizes. Real‐time prescription monitoring could detect and minimise stockpiling. Knowledge of the differences in the toxicity of medicines within a class could facilitate the prescribing of safer agents as first line medications.

## Open access

Open access publishing facilitated by the University of Sydney, as part of the Wiley – the University of Sydney agreement via the Council of Australian University Librarians.

## Competing interests

Rose Cairns has received grants from Reckitt for purposes unrelated to this study.

## Data sharing

The ASHLi dataset is accessible to named investigators only. Requests to the corresponding author for the data will require institutional approval.

## Supporting information


Supplementary methods and results

## References

[1] Zalsman G , Hawton K , Wasserman D , et al. Suicide prevention strategies revisited: 10‐year systematic review. Lancet Psychiatry 2016; 3: 646‐659. 27289303 10.1016/S2215-0366(16)30030-X

[2] Lim JS , Buckley NA , Chitty KM , et al. Association between means restriction of poison and method‐specific suicide rates: a systematic review. JAMA Health Forum 2021; 2: e213042. 35977165 10.1001/jamahealthforum.2021.3042 PMC8727039

[3] Daigle MS . Suicide prevention through means restriction: assessing the risk of substitution. A critical review and synthesis. Accid Anal Prev 2005; 37: 625‐632. 15949453 10.1016/j.aap.2005.03.004

[4] Lim M , Lee S , Park JI . Differences between impulsive and non‐impulsive suicide attempts among individuals treated in emergency rooms of South Korea. Psychiatry Investig 2016; 13: 389‐396. 10.4306/pi.2016.13.4.389 PMC4965648 27482239

[5] Lim JS , Buckley NA , Cairns R , et al. Substances detected during coroner postmortem toxicology analyses in poisoning‐ and nonpoisoning‐related suicides. JAMA Psychiatry 2023; 80: 1121‐1130. 37494023 10.1001/jamapsychiatry.2023.2289 PMC10372754

[6] Daly C , Griffin E , Corcoran P , et al. A national case fatality study of drugs taken in intentional overdose. Int J Drug Policy 2020; 76: 102609. 31884324 10.1016/j.drugpo.2019.102609

[7] Fountain JS , Tomlin AM , Reith DM , Tilyard MW . Fatal toxicity indices for medicine‐related deaths in New Zealand, 2008–2013. Drug Saf 2020; 43: 223‐232. 31749126 10.1007/s40264-019-00885-4

[8] Ojanperä I , Kriikku P , Vuori E . Fatal toxicity index of medicinal drugs based on a comprehensive toxicology database. Int J Legal Med 2016; 130: 1209‐1216. 26987318 10.1007/s00414-016-1358-8

[9] Brett J , Wylie CE , Raubenheimer J , et al. The relative lethal toxicity of pharmaceutical and illicit substances: a 16‐year study of the Greater Newcastle Hunter area, Australia. Br J Clin Pharmacol 2019; 85: 2098‐2107. 31173392 10.1111/bcp.14019 PMC6710517

[10] Miller TR , Swedler DI , Lawrence BA , et al. Incidence and lethality of suicidal overdoses by drug class. JAMA Netw Open 2020; 3: e200607. 32202643 10.1001/jamanetworkopen.2020.0607 PMC7090840

[11] Chitty KM , Buckley NA , Lim J , et al. Psychotropic and other medicine use at time of death by suicide: a population‐level analysis of linked dispensing and forensic toxicology data. Med J Aust 2023; 219: 63‐69. https://www.mja.com.au/journal/2023/219/2/psychotropic‐and‐other‐medicine‐use‐time‐death‐suicide‐population‐level‐analysis 37230472 10.5694/mja2.51985 PMC10952140

[12] Boggs JM , Simon GE , Beck A , et al. Are people who die by intentional medication poisoning dispensed those medications in the year prior to death? Arch Suicide Res 2023; 27: 1083‐1090. 35579399 10.1080/13811118.2022.2072253 PMC9762134

[13] Cairns R , Karanges EA , A Wong , et al. Trends in self‐poisoning and psychotropic drug use in people aged 5–19 years: a population‐based retrospective cohort study in Australia. BMJ Open 2019; 9: e026001. 10.1136/bmjopen-2018-026001 PMC6398641 30787095

[14] Chitty KM , Schumann JL , Schaffer A , et al. Australian Suicide Prevention using Health‐Linked Data (ASHLi): protocol for a population‐based case series study. BMJ Open 2020; 10: e038181. 10.1136/bmjopen-2020-038181 PMC7223353 32398340

[15] Australian Department of Health and Aged Care . Australian statistics on medicines 2015. Updated 18 Nov 2016. https://www.pbs.gov.au/info/statistics/asm/asm‐2015 (viewed May 2023).

[16] Mellish L , Karanges EA , Litchfield MJ , et al. The Australian Pharmaceutical Benefits Scheme data collection: a practical guide for researchers. BMC Res Notes 2015; 8: 634. 26526064 10.1186/s13104-015-1616-8 PMC4630883

[17] Huynh A , Cairns R , Brown JA , et al. Health care cost savings from Australian Poisons Information Centre advice for low risk exposure calls: SNAPSHOT 2. Clin Toxicol (Phila) 2020; 58: 752‐757. 31718323 10.1080/15563650.2019.1686513

[18] Patil V , Kulkarni H . Comparison of confidence intervals for the Poisson mean: some new aspects. Revstat Stat J 2012; 10: 211‐227.

[19] Data Commons . Australia: demographics. https://datacommons.org/place/country/AUS?category=Demographics# (viewed June 2023).

[20] World Health Organization . Anatomical Therapeutic (ATC) classification. Undated. https://www.who.int/tools/atc‐ddd‐toolkit/atc‐classification (viewed Apr 2023).

[21] Huynh A , Cairns R , Brown JA , et al. Patterns of poisoning exposure at different ages: the 2015 annual report of the Australian Poisons Information Centres. Med J Aust 2018; 209: 74‐79. https://www.mja.com.au/journal/2018/209/2/patterns‐poisoning‐exposure‐different‐ages‐2015‐annual‐report‐australian‐poisons 29976129 10.5694/mja17.01063

[22] Spicer RS , Miller TR . Suicide acts in 8 states: incidence and case fatality rates by demographics and method. Am J Public Health 2000; 90: 1885‐1891. 11111261 10.2105/ajph.90.12.1885 PMC1446422

[23] Sinyor M , Howlett A , Cheung AH , Schaffer A . Substances used in completed suicide by overdose in Toronto: an observational study of coroner's data. Can J Psychiatry 2012; 57: 184‐191. 22398005 10.1177/070674371205700308

[24] Hawton K , Bergen H , Simkin S , et al. Toxicity of antidepressants: rates of suicide relative to prescribing and non‐fatal overdose. Br J Psychiatry 2010; 196: 354‐358. 20435959 10.1192/bjp.bp.109.070219 PMC2862059

[25] Ferrey AE , Geulayov G , Casey D , et al. Relative toxicity of mood stabilisers and antipsychotics: case fatality and fatal toxicity associated with self‐poisoning. BMC Psychiatry 2018; 18: 399. 30587176 10.1186/s12888-018-1993-3 PMC6307121

[26] Buckley NA , Whyte IM , Dawson AH , Isbister GK . A prospective cohort study of trends in self‐poisoning, Newcastle, Australia, 1987–2012: plus ça change, plus c'est la même chose. Med J Aust 2015; 202: 438‐442. https://www.mja.com.au/journal/2015/202/8/prospective‐cohort‐study‐trends‐self‐poisoning‐newcastle‐australia‐1987‐2012 25929508 10.5694/mja14.01116

[27] Minns AB , Clark RF . Toxicology and overdose of atypical antipsychotics. J Emerg Med 2012; 43: 906‐913. 22555052 10.1016/j.jemermed.2012.03.002

[28] Pompili M. Adding suicide prevention to the triple advantages of injectable long‐acting second‐generation antipsychotics. Front Psychiatry 2019; 10: 931. 32009988 10.3389/fpsyt.2019.00931 PMC6971400

[29] Zhang Z , Guo L , Si R , et al. Pharmacists’ perceptions on real‐time prescription monitoring (RTPM) systems: a cross‐sectional survey. Explor Res Clin Soc Pharm 2022; 5: 100122. 35478517 10.1016/j.rcsop.2022.100122 PMC9032446

[30] Cleary E , Kelleher CC , Lane A , Malone KM . Limiting psychotropic medication prescription on discharge from psychiatric inpatient care: a possible suicide intervention? Ir J Psychol Med 2020; 37: 43‐47. 31182176 10.1017/ipm.2019.25

[31] Wong J , Motulsky A , Abrahamowicz M , et al. Off‐label indications for antidepressants in primary care: descriptive study of prescriptions from an indication based electronic prescribing system. BMJ 2017; 356: j603. 28228380 10.1136/bmj.j603 PMC5320934

[32] Darke S , Chrzanowska A , Campbell G , et al. Barbiturate‐related hospitalisations, drug treatment episodes, and deaths in Australia, 2000–2018. Med J Aust 2022; 216: 194‐198. https://www.mja.com.au/journal/2022/216/4/barbiturate‐related‐hospitalisations‐drug‐treatment‐episodes‐and‐deaths 34658038 10.5694/mja2.51306

[33] Therapeutic Goods Administration (Australian Department of Health) . Australian public assessment report for buprenorphine. Dec 2016. https://www.tga.gov.au/sites/default/files/auspar‐buprenorphine‐161213.pdf (viewed June 2023).

[34] Rock KL , Reynolds LM , Rees P , Copeland CS . Highlighting the hidden dangers of a “weak” opioid: deaths following use of dihydrocodeine in England (2001–2020). Drug Alcohol Depend 2022; 233: 109376. 35248998 10.1016/j.drugalcdep.2022.109376

[35] Schifano F , Chiappini S , Miuli A , et al. Focus on over‐the‐counter drugs’ misuse: a systematic review on antihistamines, cough medicines, and decongestants. Front Psychiatry 2021; 12: 657397. 34025478 10.3389/fpsyt.2021.657397 PMC8138162

[36] Cairns R , Schaffer AL , Brown JA , et al. Codeine use and harms in Australia: evaluating the effects of re‐scheduling. Addiction 2020; 115: 451‐459. 31577369 10.1111/add.14798

[37] 33 Therapeutic Goods Administration (Australian Department of Health and Aged Care) . DI‐GESIC tablet blister pack Cancelled under Section 30(1)(c) of the Act. 4 Apr 2018. https://www.tga.gov.au/resources/cancellations‐by‐sponsors/di‐gesic‐tablet‐blister‐pack‐cancelled‐under‐section‐301c‐act (viewed Mar 2025).

[38] Schaffer AL , Busingye D , Chidwick K , et al. Pregabalin prescribing patterns in Australian general practice, 2012–2018: a cross‐sectional study. BJGP Open 2021; 5: bjgpopen20X101120. 10.3399/bjgpopen20X101120 PMC7960512 33172853

[39] Brett J , Schaffer A , Dobbins T , et al. The impact of permissive and restrictive pharmaceutical policies on quetiapine dispensing: evaluating a policy pendulum using interrupted time series analysis. Pharmacoepidemiol Drug Saf 2018; 27: 439‐446. 29493050 10.1002/pds.4408

