# Supplementary material for: The relative toxicity of medicines detected after poisoning suicide deaths in Australia, 2013–19: a data linkage case series study
Source: Med J Aust. 2025 Mar 24;222(7):339–47. doi: 10.5694/mja2.52638 (PMC12009598; doi:10.5694/mja2.52638)
Supplement: Supplementary file 1 — Supplementary methods and results [file MJA2-222-339-s001.pdf]

Supporting Information

Supplementary methods and results

This appendix was part of the submitted manuscript and has been peer reviewed.
It is posted as supplied by the authors.

Appendix to: Lim J, Buckley NA, Chitty K, et al. The relative toxicity of medicines detected after poisoning suicide deaths in Australia, 2013–19: a data linkage case series study. *Med J Aust* 2025; doi: 10.5694/mja2.52638.

Supplementary methods

Table 1. International Statistical Classification of Diseases and Related Health Problems, Tenth Revision (ICD-10) codes for contributory medicines in medicine poisoning suicides

We extracted a list of all *International Statistical Classification of Diseases and Related Health Problems, Tenth Revision* (ICD-10) codes for poisoning (T35-T50). For each ICD-10 T code included in the National Coronial Information System coded fields of suicide death reports, we generated a list of substances that were detected with ICD-10 code. We then manually examined the list of substances for each ICD-10 code and included only substances that corresponded to the same medicine or substance class described by the ICD-10 code. We only included medicines that contributed to death in our final analysis if the ICD-10 code corresponded or the medicine was named by the coroner in the cause of death free-text field. Other substances and medicines associated with an ICD-10 code but not shown here were not detected in the medicine and drug poisoning suicides included in our study.

ICD-10 code description	ICD-10 code in cause of death	Medicine or substance considered contributory
T36: Poisoning by systemic antibiotics	T36.8	trimethoprim
T37: Poisoning by other systemic anti-infectives and antiparasitics	T37.0	sulfamethoxazole
	T37.2	quinine, chloroquine
	T37.8	hydroxychloroquine, aminochlorobenzophenone
T38: Poisoning by hormones and their synthetic substitutes and antagonists, not elsewhere classified	T38.0	prednisolone, methylprednisolone
	T38.3	insulin, metformin, gliclazide, sitagliptin
T39: Poisoning by nonopioid analgesics, antipyretics and antirheumatics	T39.0	salicylic acid
	T39.1	paracetamol
	T39.2	4-methylaminoantipyrine, dipyrone, dipyrone/4-methylaminoantipyrine
	T39.3	ibuprofen, naproxen, celecoxib, meloxicam, diclofenac, etoricoxib, indomethacin
	T39.8	paracetamol, salicylic acid
T40: Poisoning by narcotics and psychodysleptics [hallucinogens]	T40.0	laudanosine
	T40.2	codeine, morphine, 6-monoacetylcodeine, 6-monoacetylmorphine, codeine-6-glucuronide, morphine-3-glucuronide, morphine-6-glucuronide, oxycodone, tramadol, hydromorphone, fentanyl, hydrocodone, methadone, tapentadol, pholcodine, noroxycodone, EDDP (*2-Ethylidene-1,5-dimethyl-3,3-diphenylpyrrolidine)*, dextromethorphan, buprenorphine, dextropropoxyphene, pethidine, propoxyphene, dihydrocodeine
	T40.3	methadone, EDDP
	T40.4	tramadol, codeine, oxycodone, fentanyl, dextropropoxyphene, morphine, propoxyphene, buprenorphine, hydromorphone, norfentanyl, hydrocodone, pethidine, O-desmethyltramadol, noroxycodone
	T40.6	codeine, oxycodone, tapentadol, morphine
T41: Poisoning by anaesthetics and therapeutic gases	T41.0	sevoflurane
	T41.1	thiopental, pentobarbitone
	T41.2	propofol, ketamine, lidocaine/lignocaine, rocuronium
	T41.3	lidocaine/lignocaine, bupivacaine
T42: Poisoning by antiepileptic, sedative-hypnotic and antiparkinsonism drugs	T42.0	phenytoin, galantamine
	T42.1	carbamazepine
	T42.3	pentobarbitone, phenobarbitone, barbiturates
	T42.4	diazepam, temazepam, alprazolam, oxazepam, 7-aminoclonazepam, nitrazepam, 7-aminonitrazepam, clonazepam, lorazepam, midazolam, nordiazepam, bromazepam, flunitrazepam
	T42.5	lamotrigine
	T42.6	valproic acid, pregabalin, lamotrigine, topiramate, baclofen, carbamazepine, trichloroethanol
	T42.7	zopiclone, pregabalin
	T42.8	baclofen
T43: Poisoning by psychotropic drugs, not elsewhere classified	T43.0	amitriptyline, mirtazapine, dothiepin, doxepin, nortriptyline, clomipramine
	T43.1	moclobemide, tranylcypromine
	T43.2	mirtazapine, quetiapine, venlafaxine, citalopram, desmethylvenlafaxine, fluoxetine, sertraline, amitriptyline, olanzapine, duloxetine, promethazine, fluvoxamine, dothiepin, paroxetine, amisulpride, aripiprazole, nortriptyline, reboxetine, risperidone, chlorpromazine, clozapine, norfluoxetine, paliperidone, clomipramine, desmethylcitalopram, moclobemide, agomelatine, haloperidol, mianserin, vortioxetine, desmethylsertraline, desvenlafaxine, imipramine, lurasidone, tranylcypromine, ziprasidone
	T43.3	promethazine, chlorpromazine, prochlorperazine
	T43.4	haloperidol, zuclopenthixol
	T43.5	Same as T43.2
	T43.6	methylamphetamine, methylamphetamine metabolite, phentermine

ICD-10 code description	ICD-10 code in cause of death	Medicine or substance considered contributory
	T43.8	donepezil, gabapentin
T44: Poisoning by drugs primarily affecting the autonomic nervous system	T44.3	atropine, benzhexol
	T44.6	prazosin
	T44.7	propranolol, metoprolol, atenolol, bisoprolol
	T44.8	labetalol
	T44.9	ephedrine, pseudoephedrine
T45: Poisoning by primarily systemic and haematological agents, not elsewhere classified	T45.0	doxylamine, chlorpheniramine, pheniramine, cetirizine, loratadine
	T45.5	warfarin
T46: Poisoning by agents primarily affecting the cardiovascular system	T46.0	digoxin
	T46.1	amlodipine, diltiazem, verapamil, nifedipine
	T46.2	amiodarone, flecainide
	T46.3	dipyridamole
	T46.4	perindopril
	T46.5	hydrochlorothiazide, indapamide, telmisartan, valsartan
	T46.9	clopidogrel carboxylic acid, diltiazem
T47: Poisoning by agents primarily affecting the gastrointestinal system	T47.0	ranitidine
T48: Poisoning by agents primarily acting on smooth and skeletal muscles and the respiratory system	T48.1	orphenadrine
	T48.3	codeine, pholcodine, dextromethorphan
	T48.5	chlorpheniramine, pseudoephedrine
T49: Poisoning by topical agents primarily affecting skin and mucous membrane and by ophthalmological, otorhinolaryngological and dental drugs	T49.4	salicylic acid
T50: Poisoning by diuretics and other and unspecified drugs, medicaments and biological substances	T50.1	frusemide
	T50.2	hydrochlorothiazide, indapamide
	T50.3	potassium, chloride ion
	T50.4	colchicine
	T50.5	naloxone, phentermine
	T50.7	naloxone

Table 2. Metabolite and parent drug coding rules

When a metabolite and parent drug were found together, only the parent drug was counted when counting the number of agents and generating the frequency table of drugs detected.

Metabolite	Parent drug	Rules when both drugs appear together (in the same decedent)
oxazepam	temazepam	If found together, only include temazepam
oxazepam	diazepam	If found together, only include diazepam
temazepam	diazepam	If found together, only include diazepam
desmethylvenlafaxine	venlafaxine	If found together, only include venlafaxine
morphine	codeine	If found together, only include codeine
Other search terms to indicate a metabolite (in any position): metabolite, artefact, acetyl, amino, benzoyl, des, epoxide, flu, glucuronide, hydroxy, meth, mono, nor, pseudo		A metabolite was identified if contained one of the search terms. A metabolite was only excluded if a drug was present twice in the same decedent: once as the drug, and again as the same drug with a metabolite keyword

Box 1. Limitations in medicines use data

We excluded medicines from the main analysis that were unlikely to have accurate Defined Daily Dose/1000 population/day (DDD) values. These were medicines typically used for resuscitation in a hospital or ambulatory setting or medicines purchasable over-the-counter.[1]

We identified medicines where DDD was likely to be an underestimate. These were identified by searching the Pharmaceutical Benefits Scheme (PBS) online website for medicines that were only subsidised by the Repatriation Pharmaceutical Benefits Scheme (for eligible veterans and their dependants) or with an authority prescription with very specific criteria (e.g. PBS supply of quinine is restricted to treating malaria).[2] Methadone and buprenorphine supplied through the Opioid Dependence Treatment Program in hospitals and clinics were not captured in PBS medicine use data during the study period.[3]

Supplementary results

Table 3. Fatal Toxicity Index (FTI) for medicines excluded from main analysis because of non-prescription availability, resuscitation use, PBS limitations, or very small denominator (DDD in lowest 5th percentile) (graphed in figure 1)

Medicine	Suicides*	Fatal Toxicity Index (95% CI)	Dispensed FTI	Non-dispensed FTI	Category	Very small denominator†
midazolam	34	126,289 (57,590-239,082)	0 (0%)	126,289 (100%)	resuscitation	Yes
lidocaine/ lignocaine	8	70,539 (7,094-211,607)	0 (0%)	70,539 (100%)	resuscitation	Yes
pholcodine	12	40,974 (4,553-135,807)	0 (0%)	40,974 (100%)	OTC	Yes
pseudoephedrine	<6	19,376 (981-215,913)	0 (0%)	19,376 (100%)	OTC	Yes
quinine	11	5,808 (1,347-19,087)	1,089 (19%)	4,720 (81%)	PBS limitation‡	Yes
bromazepam	<6	5,768 (729-21,742)	0 (0%)	5,768 (100%)	PBS limitation§	Yes
promethazine	63	3,358 (1,922-5,460)	140 (4%)	3,218 (96%)	OTC	-
zopiclone	21	636 (269-1,378)	0 (0%)	636 (100%)	PBS limitation§	-
methadone	51	490 (312-753)	106 (22%)	384 (78%)	PBS limitation¶	-
flunitrazepam	<6	364 (12-2,588)	0 (0%)	364 (100%)	PBS limitation§	-
atropine	<6	136 (0-4,007)	0 (0%)	136 (100%)	resuscitation	-
codeine	327	104 (82.8-128)	50.0 (48%)	53.7 (52%)	OTC**	-
prochlorperazine	7	99.8 (14.5-433)	74.9 (75%)	25.0 (25%)	OTC	-
ibuprofen	43	74.4 (30.6-139)	6.42 (9%)	68.0 (91%)	OTC	-
loratadine	<6	34.0 (0-377)	0 (0%)	34.0 (100%)	OTC	-
cetirizine	<6	24.9 (0-735)	0 (0%)	24.9 (100%)	OTC	-
buprenorphine	9	24.2 (2.94-87.7)	15.6 (64%)	8.60 (36%)	PBS limitation¶	-
paracetamol	324	21.2 (17.2-26)	10.1 (47%)	11.2 (53%)	OTC	-
metoclopramide	<6	10.3 (0.19-42)	1.51 (15%)	8.79 (85%)	OTC	-
naproxen	7	2.57 (0.05-11.9)	1.25 (49%)	1.32 (51%)	OTC	-
salicylic acid	17	1.71 (0.31-4.33)	0 (0%)	1.71 (100%)	OTC	-
ranitidine	<6	1.64 (0.07-14.98)	1.64 (100%)	0 (0%)	OTC	-
diclofenac	<6	1.10 (0-12.2)	0 (0%)	1.10 (100%)	OTC	-

DDD = defined daily dose/1000 population/day; OTC = over the counter (or medicine is also available without a prescription); PBS = Pharmaceutical Benefits Scheme.

* Note: number of suicides is not the same as the scaled weights used to calculate FTI.

† Very small denominators refer to the DDD being in the lowest 5th percentile of all DDDs. These should be interpreted with caution because a very small DDD can cause an infrequently used substance to have a very high FTI.

‡ For treating for malaria only (PBS).

§ Available under Repatriation Pharmaceutical Benefits Scheme (RPBS) for eligible veteran card holders only

¶ Methadone and buprenorphine supplied through the Opioid Dependence Treatment Program in hospitals and clinics were not captured in PBS medicine use data during the study period. National changes to the supply and recording of methadone and buprenorphine occurred after the study period, on 1 July 2023.[4]

** Codeine was available OTC for most of the study period, before being made entirely prescription only in Australia from February 2018.[5]

Table 4. Fatal Toxicity Index (FTI) for prescription medicines contributory to medicine poisoning suicides, in descending order, by whether PBS recorded dispensing to the deceased person during the twelve months preceding their death (FTIs are graphed in figure 1)

Medicine	Suicide deaths*	Fatal Toxicity Index (95% CI)	Dispensed FTI	Non-dispensed FTI
Antidepressants (mean recently dispensed rate of medicines 86%)				
nortriptyline†	21	214 (101-421)	154 (72%)	59.8 (28%)
clomipramine	13	211 (77.3-459)	156 (74%)	55.6 (26%)
amitriptyline	235	190 (155-230)	155 (81%)	35.1 (19%)
dothiepin	49	164 (96.1-264)	143 (87%)	20.6 (13%)
mianserin	<6	162 (18.3-545)	75.5 (47%)	86.2 (53%)
doxepin	23	149 (69.1-265)	124 (84%)	24.1 (16%)
mirtazapine	190	63.0 (47.4-82)	55.4 (88%)	7.57 (12%)
reboxetine	7	48.2 (1.07-235)	33.7 (70%)	14.5 (30%)
fluvoxamine	18	34.1 (14.5-74.6)	34.1 (100%)	0
fluoxetine	76	28.6 (18.5-43.0)	26.4 (92%)	2.17 (8%)
venlafaxine	122	28.0 (19.8-38.0)	26.3 (94%)	1.73 (6%)
moclobemide	7	25.2 (2.59-77.2)	18.3 (73%)	6.92 (27%)
duloxetine	46	24.7 (14.02-43.0)	23.8 (96%)	0.94 (4%)
desmethylvenlafaxine†	99	18.5 (11.7-27.6)	16.2 (87%)	2.37 (13%)
imipramine	<6	13.5 (0-299)	13.5 (100%)	0
citalopram/escitalopram (78 escitalopram, 31 citalopram, 8 unknown)	117	12.0 (8.49-17.0)	11.3 (94%)	0.72 (6%)
sertraline	74	9.91 (6.51-14.9)	8.80 (89%)	1.11 (11%)
paroxetine	16	8.69 (2.99-21.5)	8.69 (100%)	0
tranylcypromine	<6	4.77 (0-123)	4.77 (100%)	0
Antipsychotics (mean recently dispensed rate of medicines 77%)				
lurasidone	<6	940 (0-7,410)	689 (73%)	251 (27%)
quetiapine	258	268 (215-332)	229 (86%)	38.8 (14%)
chlorpromazine	12	225 (69.6-654)	209 (93%)	16.0 (7%)
clozapine	15	131 (60.8-253)	97.3 (75%)	33.3 (25%)
amisulpride	13	90.2 (24.0-225)	90.2 (100%)	0
olanzapine	97	78.3 (51.7-111)	65.0 (83%)	13.3 (17%)
haloperidol	7	64.5 (9.77-291)	35.0 (54%)	29.6 (46%)
zuclopenthixol	<6	59.3 (8.21-245)	0	59.3 (100%)
aripiprazole	13	37.4 (6.98-98.9)	30.7 (82%)	6.69 (18%)
risperidone	12	16.1 (3.97-56.2)	13.1 (81%)	3.00 (19%)
paliperidone	6	11.7 (0.31-67.3)	8.67 (74%)	3.02 (26%)
lithium	<6	6.77 (0.17-36.5)	6.77 (100%)	0
ziprasidone	<6	6.40 (0-165)	6.40 (100%)	0
Hypnosedatives (mean recently dispensed rate of medicines 59%)				
clonazepam	34	1,592 (1,020-2,368)	250 (16%)	1,342 (84%)
diazepam	467	201 (169-237)	151 (75%)	50.0 (25%)
nitrazepam	71	172 (111-263)	104 (61%)	68.0 (39%)
temazepam†	150	141 (107-184)	112 (79%)	29.2 (21%)
oxazepam†	71	130 (84.9-194)	96.8 (74%)	33.7 (26%)
alprazolam	91	77.4 (50.1-112)	35.7 (46%)	41.7 (54%)
Opioids (mean recently dispensed rate of medicines 53%)				
oxycodone	385	365 (309-431)	233 (64%)	132 (36%)
morphine†	79	241 (163-344)	67.5 (28%)	173 (72%)

Medicine	Suicide deaths*	Fatal Toxicity Index (95% CI)	Dispensed FTI	Non-dispensed FTI
hydromorphone	28	211 (103-394)	95.6 (45%)	115 (55%)
fentanyl	58	163 (105-244)	86.7 (53%)	76.5 (47%)
tapentadol	12	143 (31.4-445)	96.7 (67%)	46.6 (33%)
tramadol	141	106 (75.7-143)	63.2 (59%)	43.1 (41%)
Antiepileptics (mean recently dispensed rate of medicines 61%)				
phenobarbitone	14	677 (279-1,429)	0	677 (100%)
topiramate	7	32.5 (4.56-136)	27.3 (84%)	5.23 (16%)
lamotrigine	8	31.9 (9.7-91.4)	20.7 (65%)	11.2 (35%)
carbamazepine	16	30.3 (12.0-71.5)	20.7 (68%)	9.63 (32%)
valproic acid	33	26.0 (12.5-44.6)	24.9 (96%)	1.02 (4%)
phenytoin	<6	24.4 (5.11-72.4)	20.2 (83%)	4.13 (17%)
pregabalin	66	23.3 (12.0-38.5)	20.6 (88%)	2.68 (12%)
gabapentin	<6	13.5 (0-110)	0	13.5 (100%)
Other psychotropic and non-psychotropic medicines (mean recently dispensed rate of medicines 72%)				
propranolol	71	204 (139-293)	168 (82%)	36.1 (18%)
atomoxetine	<6	76.8 (1.94-428)	76.8 (100%)	0
bupropion	<6	58.3 (0-646)	0	58.3 (100%)
baclofen	17	57.7 (19.5-140)	48.4 (84%)	9.39 (16%)
labetalol	<6	30.0 (1.5-334.8)	0	30.0 (100%)
hydroxychloroquine	13	27.8 (7.97-74.9)	22.7 (82%)	5.12 (18%)
sulfamethoxazole	<6	27.5 (0-508)	0	27.5 (100%)
benztropine	<6	27.3 (0.52-114)	10.2 (38%)	17.1 (63%)
methylprednisolone	<6	18.5 (0-410)	0	18.5 (100%)
metoprolol	39	13.9 (7.88-23.2)	11.2 (81%)	2.64 (19%)
diltiazem	18	13.5 (5.65-29.0)	12.9 (96%)	0.54 (4%)
verapamil	13	11.9 (4.18-30.1)	8.14 (69%)	3.74 (31%)
flecainide	<6	11.0 (0.27-59.3)	8.88 (81%)	2.13 (19%)
insulin	28	8.31 (5.05-12.5)	5.05 (61%)	3.27 (39%)
digoxin	<6	7.68 (1.78-25.3)	7.68 (100%)	0
atenolol	16	4.37 (1.41-10.1)	3.76 (86%)	0.61 (14%)
bisoprolol	<6	4.06 (0.12-26.1)	4.06 (100%)	0
galantamine	<6	4.03 (0-104)	4.03 (100%)	0
amiodarone	<6	4.00 (0.19-41.1)	2.77 (69%)	1.23 (31%)
prazosin	<6	3.29 (0.07-15.7)	3.29 (100%)	0
potassium	<6	3.05 (0.15-33.9)	0	3.05 (100%)
dipyridamole	<6	3.01 (0.13-28.7)	1.29 (43%)	1.72 (57%)
metformin	24	2.90 (1.28-5.84)	2.86 (98%)	0.05 (2%)
colchicine	<6	2.19 (0-32.4)	0	2.19 (100%)
amlodipine	22	1.79 (0.75-3.42)	1.32 (74%)	0.47 (26%)
donepezil	<6	1.78 (0-26.2)	1.78 (100%)	0
gliclazide	6	1.65 (0.36-5.06)	1.38 (83%)	0.27 (17%)
warfarin	<6	1.48 (0.04-8.29)	1.48 (100%)	0
celecoxib	<6	1.41 (0.04-8.26)	1.41 (100%)	0
meloxicam	<6	0.81 (0.03-6.45)	0.52 (64%)	0.29 (36%)
trimethoprim	<6	0.79 (0-14.6)	0.79 (100%)	0
frusemide	7	0.69 (0.11-3.27)	0.47 (67%)	0.23 (33%)
indapamide	<6	0.69 (0-5.54)	0.69 (100%)	0
nifedipine	<6	0.69 (0-10.1)	0.69 (100%)	0
sotalol	<6	0.62 (0-13.8)	0	0.62 (100%)

Medicine	Suicide deaths*	Fatal Toxicity Index (95% CI)	Dispensed FTI	Non-dispensed FTI
prednisolone	<6	0.51 (0.02-4.08)	0.37 (71%)	0.15 (29%)
hydrochlorothiazide	<6	0.51 (0.01-2.39)	0.51 (100%)	0
sitagliptin	<6	0.48 (0-8.87)	0.48 (100%)	0
valsartan	<6	0.41 (0-3.32)	0.41 (100%)	0
telmisartan	<6	0.19 (0.01-1.62)	0.19 (100%)	0
irbesartan	<6	0.17 (0.01-1.17)	0.15 (85%)	0.03 (15%)
perindopril	<6	0.09 (0.004-0.97)	0.09 (100%)	0
**Class summaries**				
Opioids	763	221 (196-248)		
Hypnosedatives	969	170 (152-191)		
Antipsychotics	444	104 (87.2-122)		
Antidepressants	1120	31.4 (28.3-34.7)		
Antiepileptics	151	30.0 (22.0-40.0)		
Other psychotropic and non-psychotropic medicines	347	3.66 (3.07-4.35)		
All classes		32.0 (30.6-33.3)		

* Note: number of suicides is not the same as the scaled weights used to calculate FTI.

† Only included if not as metabolite together with parent compound.

Figure 1. Fatal toxicity index (FTI) values for prescription medicines implicated in medicine poisoning suicide deaths

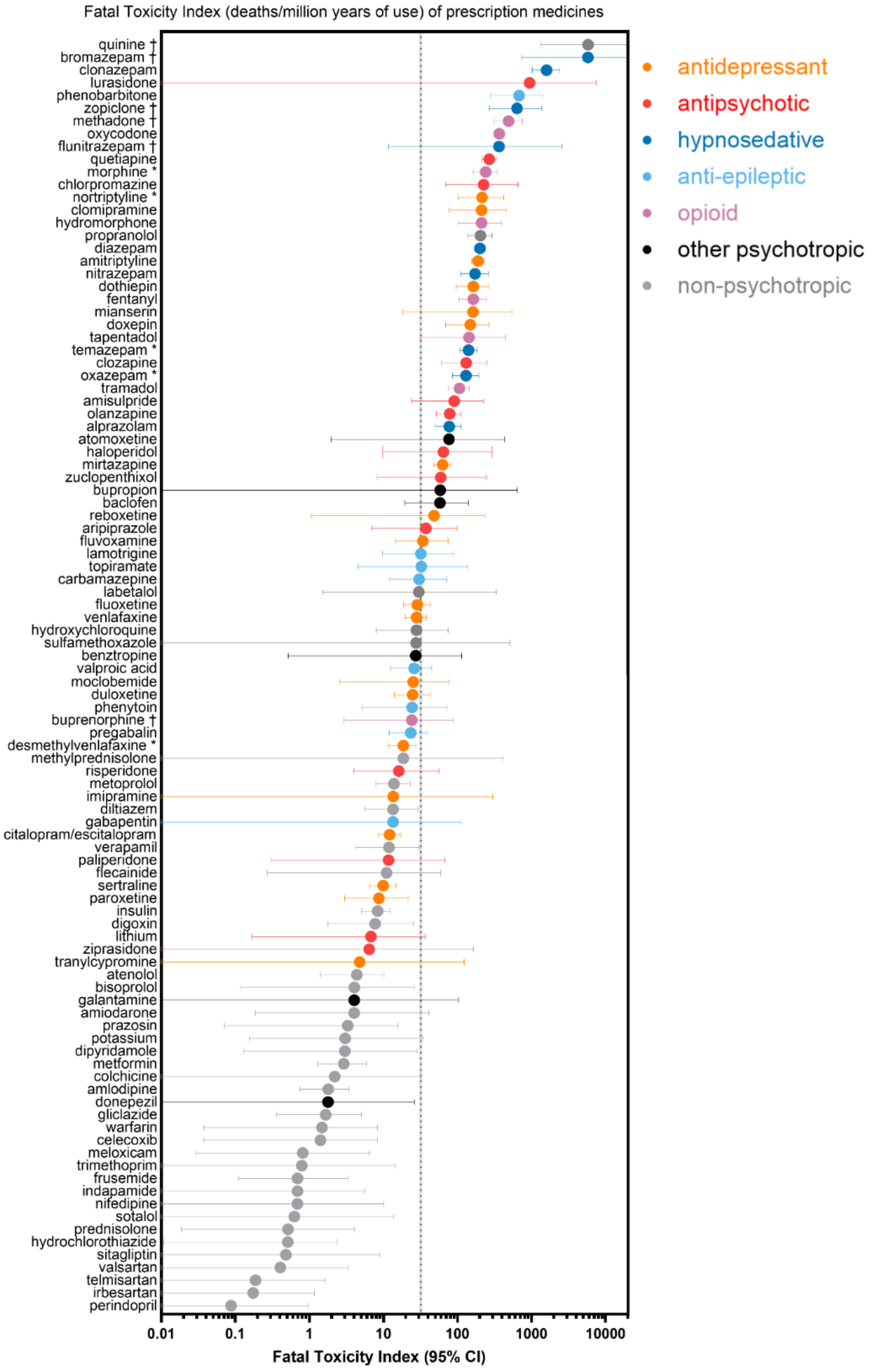

CI = confidence interval.

Deaths were scaled, where the weighted value of each medicine was adjusted according to the number of contributory medicines for each decedent. Dotted line represents overall estimated case fatality for all deaths (95% CI in shaded area).

* Included only if parent compound was not also detected (Supporting Information, table 2).

† Should be interpreted with caution as population use (DDD) is probably underestimated, leading to a higher FTI; their use in clinics or hospitals is not captured by the PBS (methadone, buprenorphine) or PBS subsidisation is restricted (eg quinine is subsidised for malaria only, bromazepam and zopiclone are subsidised only for veterans).

Table 5. Fatal toxicity index (FTI) for other nervous system medicines according to Anatomical Therapeutic Chemical (ATC) Classification[6] that were not detected and contributory

These medicines had FTIs of 0 but had a published DDD which we used to calculate upper 95% confidence interval (CI). A lower upper 95% CI suggests this medicine was not detected in suicides in our study but their population use is greater than for drugs with a higher upper 95% CI value.

Medicine	Fatal toxicity index (95% CI)
methylphenidate	0 (0-11.7)
dexamfetamine	0 (0-17.3)
varenicline	0 (0-23.1)
levetiracetam	0 (0-24.2)
pizotifen	0 (0-47.7)
methyldopa	0 (0-56.3)
pramipexole	0 (0-60.9)
rasagiline	0 (0-67.0)
rivastigmine	0 (0-119)
fluphenazine	0 (0-143)
flupentixol	0 (0-150)
pyridostigmine	0 (0-192)
rizatriptan	0 (0-200)
acamprosate	0 (0-237)
naltrexone	0 (0-243)
memantine	0 (0-284)
amantadine	0 (0-287)
eletriptan	0 (0-296)
lacosamide	0 (0-299)
oxcarbazepine	0 (0-318)
lisdexamfetamine	0 (0-346)
asenapine	0 (0-358)
zonisamide	0 (0-364)
primidone	0 (0-392)
periciazine	0 (0-395)
modafinil	0 (0-418)
selegiline	0 (0-449)
zolmitriptan	0 (0-535)
trifluoperazine	0 (0-588)
apomorphine	0 (0-588)
levodopa	0 (0-618)
rotigotine	0 (0-669)
biperiden	0 (0-681)
vigabatrin	0 (0-780)
riluzole	0 (0-840)
naratriptan	0 (0-840)
phenelzine	0 (0-960)
ethosuximide	0 (0-981)
tetrabenazine	0 (0-1,079)
levodopa and decarboxylase inhibitor	0 (0-1,090)
bethanechol	0 (0-1,179)
tiagabine	0 (0-1,330)
methysergide	0 (0-1,368)

Medicine	Fatal toxicity index (95% CI)
perampanel	0 (0-2,106)
buspirone	0 (0-4,862)

Table 6. Estimated case fatality for medicines excluded from main analysis because of resuscitation use or very small denominators (New South Wales Poisons Information Centre calls for self-poisoning in lowest 5th percentile) (graphed in figure 2)

Medicine	Number of suicides*	Estimated case fatality (95% confidence interval)	Category	Very small denominator†
pethidine	<6	93.3 (12.1-361)		Y
midazolam	34	45.1 (20.6-85)	resuscitation drug	Y
chloroquine	<6	29.2 (9.08-85.3)		Y
lidocaine/lignocaine	8	24.1 (2.42-72.2)	resuscitation drug	-
etoricoxib	<6	10.0 (0-184)		Y
dipyridamole	<6	9.72 (0.42-92.9)		Y
ephedrine	<6	8.89 (0.42-92.9)		Y
galantamine	<6	7.14 (0-184)		Y
ketamine	<6	0.81 (0.03-7.33)	resuscitation drug	-
atropine	<6	0.42 (0-12.3)	resuscitation drug	-

* Note: number of suicides is not the same as the scaled weights used to calculate estimated case fatality

† Very small denominators refer to the number of Poisons Information Centre calls being in the lowest 5th percentile of all calls. These should be interpreted with caution because a very small number of calls can cause an uncommon substance to have a very high estimated case fatality.

Table 7. Estimated case fatality for medicines contributory to medicine poisoning suicides in descending order (graphed in figure 2)

Medicine	Number of suicides*	Estimated case fatality (95% confidence interval)
Antidepressants		
doxepin	23	7.25 (3.38-13.0)
dothiepin	49	7.08 (4.16-11.4)
nortriptyline†	21	5.64 (2.67-11.1)
mianserin	<6	4.46 (0.50-15.1)
amitriptyline	235	4.05 (3.30-4.92)
clomipramine	13	3.62 (1.33-7.87)
moclobemide	7	2.46 (0.25-7.53)
reboxetine	7	1.91 (0.04-9.29)
mirtazapine	190	1.55 (1.17-2.02)
vortioxetine	<6	1.40 (0.06-12.1)
venlafaxine	122	1.36 (0.96-1.84)
desmethylvenlafaxine†	99	0.99 (0.63-1.48)
duloxetine	46	0.81 (0.46-1.41)
citalopram/escitalopram (78 escitalopram, 31 citalopram, 8 unknown)	117	0.77 (0.54-1.08)
fluvoxamine	18	0.70 (0.30-1.53)
sertraline	74	0.62 (0.41-0.94)
paroxetine	16	0.57 (0.20-1.41)
fluoxetine	76	0.52 (0.37-0.85)
tranylcypromine	<6	0.51 (0-13.2)
imipramine	<6	0.46 (0-10.2)
Antipsychotics		
zuclopenthixol	<6	7.95 (1.10-32.8)
clozapine	15	2.91 (1.35-5.62)
amisulpride	13	1.72 (0.46-4.30)
haloperidol	7	1.23 (0.19-5.56)
olanzapine	97	0.93 (0.62-1.32)
paliperidone	6	0.83 (0.02-4.80)
quetiapine	258	0.66 (0.53-0.81)
aripiprazole	13	0.60 (0.11-1.58)
promethazine	63	0.54 (0.31-0.88)
prochlorperazine	7	0.51 (0.07-2.19)
lurasidone	<6	0.36 (0-2.84)
chlorpromazine	12	0.33 (0.10-0.97)
risperidone	12	0.23 (0.06-0.81)
ziprasidone	<6	0.09 (0-2.33)
lithium	<6	0.08 (0.002-0.41)
Hypnosedatives		
nitrazepam	71	4.53 (2.92-6.90)
bromazepam	<6	2.52 (0.32-9.51)
clonazepam	91	1.79 (1.14-2.66)
oxazepam†	71	1.60 (1.04-2.37)
temazepam†	150	1.34 (1.01-1.75)
diazepam	467	1.26 (1.06-1.48)
alprazolam	91	1.08 (0.70-1.57)

Medicine	Number of suicides*	Estimated case fatality (95% confidence interval)
lorazepam	35	0.90 (0.40-1.82)
doxylamine	87	0.82 (0.53-1.22)
flunitrazepam	<6	0.74 (0.02-5.26)
zopiclone	21	0.58 (0.25-1.26)
zolpidem	13	0.47 (0.12-1.08)
Antiepileptics		
phenobarbitone	14	12.2 (5.03-25.8)
phenytoin	<6	2.42 (0.51-7.19)
carbamazepine	16	0.72 (0.29-1.70)
topiramate	7	0.57 (0.08-2.38)
pregabalin	66	0.49 (0.25-0.81)
lamotrigine	8	0.45 (0.14-1.28)
valproic acid	33	0.44 (0.21-0.75)
gabapentin	<6	0.17 (0-1.37)
Opioids		
dextropropoxyphene	16	23.3 (7.38-53.0)
fentanyl	58	12.3 (7.93-18.4)
hydromorphone	28	12.3 (5.99-23.0)
morphine†	79	10.3 (6.93-14.7)
methadone	51	5.45 (3.46-8.37)
codeine	327	4.70 (3.75-5.78)
pholcodine	12	3.11 (0.35-10.3)
oxycodone	385	2.75 (2.33-3.24)
tramadol	141	1.96 (1.39-2.63)
tapentadol	12	1.16 (0.25-3.59)
buprenorphine	9	0.74 (0.09-2.70)
dihydrocodeine	<6	0.55 (0 -4.39)
Other psychotropic and non-psychotropic medicines		
quinine	11	11.1 (2.58-36.5)
hydroxychloroquine	13	7.05 (2.02-19.0)
diltiazem	18	6.21 (2.61-13.4)
flecainide	<6	4.31 (0.11-23.2)
verapamil	13	3.78 (1.33-9.56)
salicylic acid	17	3.46 (0.62-8.77)
insulin	28	3.40 (2.07-5.11)
propranolol	71	3.06 (2.07-4.39)
amiodarone	<6	3.01 (0.14-31.0)
labetalol	<6	2.78 (0.14-31.0)
metoprolol	39	2.74 (1.56-4.60)
digoxin	<6	2.61 (0.61-8.60)
orphenadrine	<6	2.38 (0.05-9.95)
atenolol	16	2.24 (0.72-5.21)
amlodipine	22	1.82 (0.76-3.47)
indapamide	<6	1.76 (0-14.2)
gliclazide	6	1.58 (0.34-4.82)
hydrochlorothiazide	<6	1.49 (0.03-6.96)
diphenhydramine	<6	1.33 (0.16-4.82)
atomoxetine	<6	1.25 (0.03-6.96)
pheniramine	<6	1.25 (0.05-11.1)

Medicine	Number of suicides*	Estimated case fatality (95% confidence interval)
bisoprolol	<6	1.24 (0.04-7.96)
baclofen	17	1.16 (0.39-2.80)
metformin	24	0.93 (0.41-1.88)
donepezil	<6	0.83 (0.00-12.3)
frusemide	7	0.82 (0.13-3.88)
valsartan	<6	0.75 (0-6.15)
benztropine	<6	0.74 (0.01-3.10)
dextromethorphan	9	0.66 (0.09-2.64)
nifedipine	<6	0.60 (0-8.78)
sulfamethoxazole	<6	0.59 (0-10.8)
bupropion	<6	0.56 (0-6.15)
benzhexol	<6	0.52 (0-11.5)
warfarin	<6	0.46 (0.01-2.60)
prazosin	<6	0.43 (0.01-2.05)
ranitidine	<6	0.40 (0.02-3.67)
irbesartan	<6	0.39 (0.01-2.60)
celecoxib	<6	0.35 (0.01-2.03)
paracetamol	324	0.32 (0.26-0.40)
pseudoephedrine	<6	0.29 (0.01-3.24)
sotalol	<6	0.27 (0-5.95)
colchicine	<6	0.24 (0-3.55)
sitagliptin	<6	0.23 (0-4.19)
telmisartan	<6	0.23 (0.01-1.95)
metoclopramide	<6	0.22 (0.004-0.90)
caffeine	<6	0.21 (0.03-1.02)
naproxen	7	0.21 (0.004-0.98)
trimethoprim	<6	0.19 (0-3.55)
phentermine	<6	0.19 (0.01-1.63)
meloxicam	<6	0.19 (0.01-1.50)
pericyazine	<6	0.18 (0-1.50)
prednisolone	<6	0.18 (0.01-1.44)
naloxone	9	0.16 (0.02-0.68)
loratadine	<6	0.11 (0-1.21)
ibuprofen	43	0.09 (0.04-0.17)
indomethacin	<6	0.08 (0-2.12)
agomelatine	<6	0.08 (0-0.87)
cetirizine	<6	0.05 (0-1.49)
diclofenac	<6	0.05 (0-0.50)
**Class summaries**		
Opioids	763	3.12 (2.76-3.51)
Other psychotropic and non-psychotropic medicines	347	1.67 (1.40-1.98)
Hypnosedatives	969	1.35 (1.20-1.51)
Antidepressants	1120	1.32 (1.19-1.46)
Antipsychotics	444	0.67 (0.57-0.79)
Antiepileptics	151	0.60 (0.44-0.80)
All classes		1.28 (1.23-1.34)

* Number of suicides is not the same as the scaled weights used to calculate estimated case fatality.

† Only included if not as metabolite together with parent compound.

Figure 2. Estimated case fatality for the one hundred medicines implicated in medicine poisoning suicide deaths with highest case fatality values

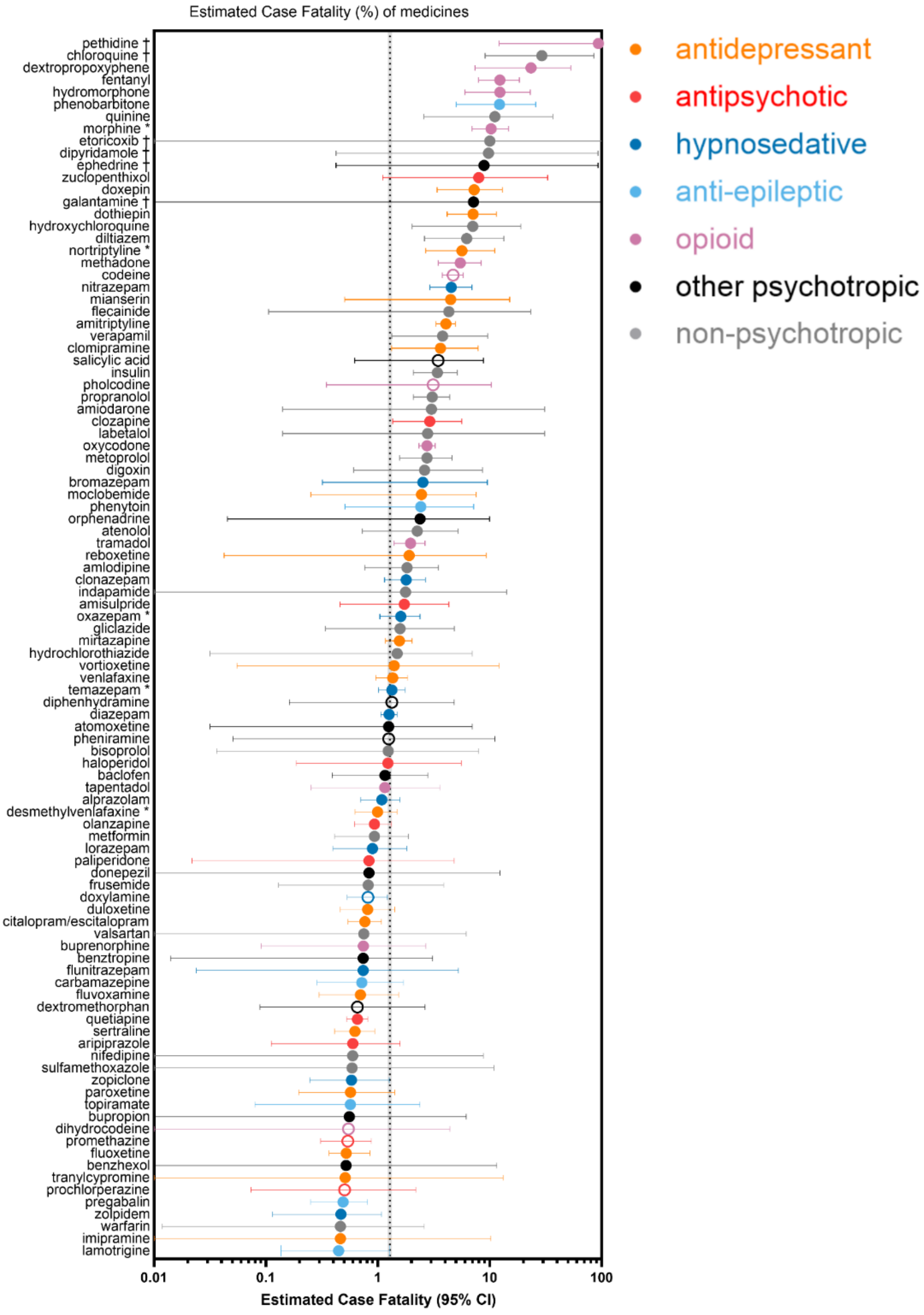

CI = confidence interval.

Deaths were scaled, where the weighted value of each medicine was adjusted according to the number of contributory medicines for each decedent. An open circle indicates medicines that are also available OTC or without prescription; these medicines have an estimated case fatality but no FTI. Dotted line represents overall estimated case fatality for all deaths (95% CI in shaded area).

* Only included if not as metabolite together with parent compound.

† Should be interpreted with caution as number of calls regarding intentional self-poisoning was very small (Poisons Information Centre calls in the lowest 5th percentile), possibly inflating case fatality estimate.

Figure 3. Scatter plot of fatal toxicity index and estimated case fatality for all medicines*

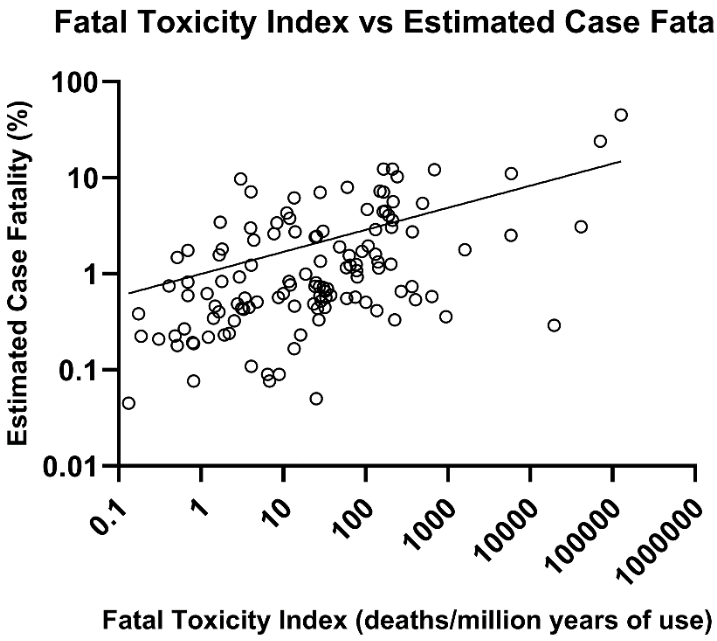

* Simple linear regression equation was calculated as estimated case fatality=0.00031*FTI + 2.016. (R^2=0.66). Differences between these measures are likely to be explained by the frequency of substances being taken in overdose. Drugs with a relatively higher FTI than expected from their case fatality are probably ingested at higher rates e.g. bottom right. Conversely, those above the line are likely to be infrequently taken but have high toxicity when ingested.

References

1 Pharmaceutical Benefits Scheme. Australian statistics on medicines 2011. Updated 28 Jan 2014. https://www.pbs.gov.au/info/statistics/asm/asm-2011 (viewed May 2023).

2 Australian Department of Health and Aged Care. Pharmaceutical Benefits Scheme: A-Z medicine listing: viewing by Drug. https://www.pbs.gov.au/browse/medicine-listing (viewed June 2023).

3 Australian Department of Health and Aged Care. Australian statistics on medicines 2015. Updated 18 Nov 2016. https://www.pbs.gov.au/info/statistics/asm/asm-2015 (viewed May 2023).

4 New South Wales Health: Centre for Alcohol and Other drugs. Opioid Treatment Program – Transition to Section 100 Highly Specialised Drugs Program. Updated 15 Sep 2023. https://www.health.nsw.gov.au/aod/pages/otp-transition-s100-hsd-program.aspx. (viewed Aug 2024)

5 Cairns R, Schaffer AL, Brown JA, et al. Codeine use and harms in Australia: evaluating the effects of re-scheduling. *Addiction* 2020; 115: 451-459.

6 World Health Organization. Anatomical Therapeutic (ATC) classification. Undated. https://www.who.int/tools/atc-ddd-toolkit/atc-classification (viewed Apr 2023).

STrengthening the Reporting of OBservational studies in Epidemiology (STROBE) Statement—checklist of items that should be included in reports of observational studies

Note: The numbers refer to the submitted manuscript, not to the published article or its supporting information file.

	**Item No**	**Recommendation**	**Page No**
**Title and abstract**	1	(*a*) Indicate the study's design with a commonly used term in the title or the abstract	1-2
		(*b*) Provide in the abstract an informative and balanced summary of what was done and what was found	2
Introduction			
Background/rationale	2	Explain the scientific background and rationale for the investigation being reported	4
Objectives	3	State specific objectives, including any prespecified hypotheses	4-5
Methods			
Study design	4	Present key elements of study design early in the paper	5-6
Setting	5	Describe the setting, locations, and relevant dates, including periods of recruitment, exposure, follow-up, and data collection	5-7
Participants	6	(*a*) *Cohort study*—Give the eligibility criteria, and the sources and methods of selection of participants. Describe methods of follow-up<br>*Case-control study*—Give the eligibility criteria, and the sources and methods of case ascertainment and control selection. Give the rationale for the choice of cases and controls<br>*Cross-sectional study*—Give the eligibility criteria, and the sources and methods of selection of participants	5-7
		(*b*) *Cohort study*—For matched studies, give matching criteria and number of exposed and unexposed<br>*Case-control study*—For matched studies, give matching criteria and the number of controls per case	
Variables	7	Clearly define all outcomes, exposures, predictors, potential confounders, and effect modifiers. Give diagnostic criteria, if applicable	6-7
Data sources/ measurement	8*	For each variable of interest, give sources of data and details of methods of assessment (measurement). Describe comparability of assessment methods if there is more than one group	6-7
Bias	9	Describe any efforts to address potential sources of bias	6-7
Study size	10	Explain how the study size was arrived at	8, Fig 1
Quantitative variables	11	Explain how quantitative variables were handled in the analyses. If applicable, describe which groupings were chosen and why	6-7
Statistical methods	12	(*a*) Describe all statistical methods, including those used to control for confounding	6-7
		(*b*) Describe any methods used to examine subgroups and interactions	5-7
		(*c*) Explain how missing data were addressed	8-9, 10-11
		(*d*) *Cohort study*—If applicable, explain how loss to follow-up was addressed<br>*Case-control study*—If applicable, explain how matching of cases and controls was addressed<br>*Cross-sectional study*—If applicable, describe analytical methods taking account of sampling strategy	
		(*e*) Describe any sensitivity analyses	
Results			
Participants	13*	(a) Report numbers of individuals at each stage of study—eg numbers potentially eligible, examined for eligibility, confirmed eligible, included in the study, completing follow-up, and analysed	8
		(b) Give reasons for non-participation at each stage	
		(c) Consider use of a flow diagram	12
Descriptive data	14*	(a) Give characteristics of study participants (eg demographic, clinical, social) and information on exposures and potential confounders	5

		(b) Indicate number of participants with missing data for each variable of interest	16, 19
		(c) *Cohort study*—Summarise follow-up time (eg, average and total amount)	
Outcome data	15*	*Cohort study*—Report numbers of outcome events or summary measures over time	8-9
		Case-control study—Report numbers in each exposure category, or summary measures of exposure	
		Cross-sectional study—Report numbers of outcome events or summary measures	
Main results	16	(*a*) Give unadjusted estimates and, if applicable, confounder-adjusted estimates and their precision (eg, 95% confidence interval). Make clear which confounders were adjusted for and why they were included	19-22
		(*b*) Report category boundaries when continuous variables were categorized	
		(*c*) If relevant, consider translating estimates of relative risk into absolute risk for a meaningful time period	
Other analyses	17	Report other analyses done—eg analyses of subgroups and interactions, and sensitivity analyses	8-9
Discussion			
Key results	18	Summarise key results with reference to study objectives	8-9
Limitations	19	Discuss limitations of the study, taking into account sources of potential bias or imprecision. Discuss both direction and magnitude of any potential bias	11-13
Interpretation	20	Give a cautious overall interpretation of results considering objectives, limitations, multiplicity of analyses, results from similar studies, and other relevant evidence	9-10, 11-12
Generalisability	21	Discuss the generalisability (external validity) of the study results	10-12
Other information			
Funding	22	Give the source of funding and the role of the funders for the present study and, if applicable, for the original study on which the present article is based	5

*Give information separately for cases and controls in case-control studies and, if applicable, for exposed and unexposed groups in cohort and cross-sectional studies.

Note: An Explanation and Elaboration article discusses each checklist item and gives methodological background and published examples of transparent reporting. The STROBE checklist is best used in conjunction with this article (freely available on the Web sites of PLoS Medicine at http://www.plosmedicine.org/, Annals of Internal Medicine at http://www.annals.org/, and Epidemiology at http://www.epidem.com/). Information on the STROBE Initiative is available at www.strobe-statement.org.
